# 4-Amino-3-{1-[4-(2-methyl­prop­yl)phen­yl]eth­yl}-1*H*-1,2,4-triazole-5(4*H*)-thione

**DOI:** 10.1107/S1600536808022794

**Published:** 2008-07-26

**Authors:** Hoong-Kun Fun, K. V. Sujith, P. S. Patil, B. Kalluraya, Suchada Chantrapromma

**Affiliations:** a X-ray Crystallography Unit, School of Physics, Universiti Sains Malaysia, 11800 USM, Penang, Malaysia; b Department of Studies in Chemistry, Mangalore University, Mangalagangotri, Mangalore 574 199, India; c Department of Studies in Physics, Mangalore University, Mangalagangotri, Mangalore 574 199, India; d Crystal Materials Research Unit, Department of Chemistry, Faculty of Science, Prince of Songkla University, Hat-Yai, Songkhla 90112, Thailand

## Abstract

In the title triazole compound, C_14_H_20_N_4_S, the dihedral angle between the triazole and benzene rings is 83.29 (11)°. The methine H atom and two methyl groups of the isobutyl group are disordered over two sites with occupancies of 0.684 (9) and 0.316 (9). In the crystal structure, N—H⋯S hydrogen bonds link the mol­ecules into chains running along the *b* axis. These chains are cross-linked into a two-dimensional network parallel to the *ab* plane by C—H⋯S hydrogen bonds.

## Related literature

For bond-length data, see: Allen *et al.* (1987 ▶). For related structures, see: Fun *et al.* (2008*a* ▶,*b* ▶,*c* ▶). For the activities and applications of 1,2,4-triazole derivatives, see: Bhat *et al.* (2004 ▶); Holla *et al.* (2002 ▶); Karthikeyan *et al.* (2007 ▶); Raafat *et al.* (2006 ▶); Wei *et al.* (2007 ▶).
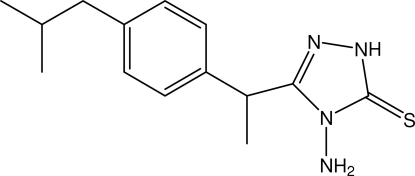


## Experimental

### 

#### Crystal data

C_14_H_20_N_4_S
*M* *_r_* = 276.41
Monoclinic, 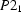

*a* = 5.9720 (3) Å
*b* = 8.5153 (5) Å
*c* = 14.8271 (6) Å
β = 97.223 (3)°
*V* = 748.03 (7) Å^3^
*Z* = 2
Mo *K*α radiation
μ = 0.21 mm^−1^
*T* = 100.0 (1) K
0.58 × 0.39 × 0.13 mm

#### Data collection

Bruker SMART APEXII CCD area-detector diffractometer
Absorption correction: multi-scan (*SADABS*; Bruker, 2005 ▶) *T*_min_ = 0.888, *T*_max_ = 0.974
10373 measured reflections
3612 independent reflections
3295 reflections with *I* > 2σ(*I*)
*R*_int_ = 0.029

#### Refinement

*R*[*F* ^2^ > 2σ(*F* ^2^)] = 0.045
*wR*(*F* ^2^) = 0.119
*S* = 1.07
3612 reflections
199 parameters
29 restraints
H atoms treated by a mixture of independent and constrained refinement
Δρ_max_ = 0.74 e Å^−3^
Δρ_min_ = −0.54 e Å^−3^
Absolute structure: Flack (1983 ▶), 1293 Friedel pairs
Flack parameter: 0.05 (9)

### 

Data collection: *APEX2* (Bruker, 2005 ▶); cell refinement: *APEX2*; data reduction: *SAINT* (Bruker, 2005 ▶); program(s) used to solve structure: *SHELXTL* (Sheldrick, 2008 ▶); program(s) used to refine structure: *SHELXTL*; molecular graphics: *SHELXTL*; software used to prepare material for publication: *SHELXTL* and *PLATON* (Spek, 2003 ▶).

## Supplementary Material

Crystal structure: contains datablocks global, I. DOI: 10.1107/S1600536808022794/ci2633sup1.cif

Structure factors: contains datablocks I. DOI: 10.1107/S1600536808022794/ci2633Isup2.hkl

Additional supplementary materials:  crystallographic information; 3D view; checkCIF report

## Figures and Tables

**Table 1:** Hydrogen-bond geometry (Å, °)

*D*—H⋯*A*	*D*—H	H⋯*A*	*D*⋯*A*	*D*—H⋯*A*
N1—H1⋯S1^i^	0.88	2.45	3.272 (3)	155
N4—H4*A*⋯S1^ii^	0.85 (3)	2.54 (3)	3.392 (3)	176 (3)
C5—H5⋯S1^iii^	0.95	2.78	3.704 (2)	165

Symmetry codes: (i) 
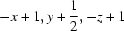
; (ii) 
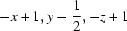
; (iii) 
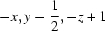
.

**Fig. 1.**
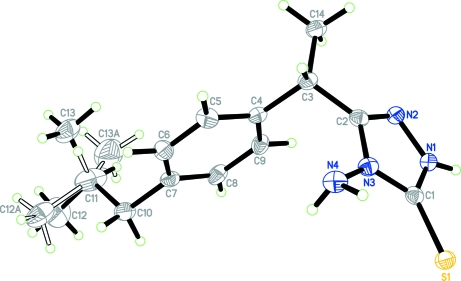
The molecular structure of the title compound, showing 50% probability displacement ellipsoids and the atom-numbering scheme. Both disorder components are shown.

**Fig. 2.**
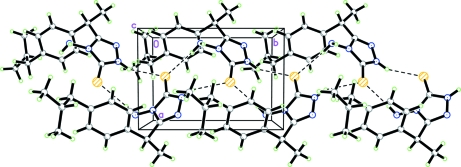
The packing diagram of the title compound, viewed along the c axis. Only the major disorder component is shown. Hydrogen bonds are shown as dashed lines.

## supplementary crystallographic information

### Comment

In recent decades, a large number of reports concerning 4-amino-1,2,4-triazol-3-thiones have appeared owing to a wide variety of their biological activity. The 1,2,4-triazole nucleus has been incorporated into a wide variety of therapeutically interesting drug candidates, including H1/H2 histamine receptor blockers, cholinesterase-active agents, *CNS* stimulants, antianxiety agents, and sedatives (Bhat *et al.*, 2004). The amino and thione groups are ready-made nucleophilic centers for the synthesis of condensed nitrogen and sulfur heterocyclic rings, *e.g.*, triazolothiadiazoles, triazolothiadiazines and triazolothiadiazepines (Raafat *et al.*, 2006). Substituted derivatives of triazole possess comprehensive bioactivities such as antimicrobial, anti-inflammatory, analgesic, antitumorial, antihypertensive, anticonvulsant and antiviral activities (Wei *et al.*, 2007). The broad biological activities that the 1,2,4-triazoles shown may be due to the presence of the >N—C—S moiety (Holla *et al.*, 2002). Due to the progress that occurs in dealing with the chemistry of substituted 4-amino-1,2,4-triazole-3-thiones as well as their biological activity, we report here the crystal structure of the title triazole compound.

The bond distances and angles in the title molecule (Fig .1) have normal values (Allen *et al.*, 1987) and are comparable with those observed in related structures (Fun *et al.*, 2008*a*,*b*,*c*). The triazole ring (C1/C2/N1-N3) is planar to within ±0.004 Å. The chiral carbon atom C3 is in a distorted tetrahedral configuration. The dihedral angle between the triazole and benzene (C4-C9) rings is 83.29 (11)°. The 2-methylpropyl group is disordered over two sites. The orientation of this group with respect to the benzene ring can be indicated by the torsion angles C7–C10–C11–C12 = 167.1 (4)° and C7–C10–C11–C13 = -53.8 (5)° [C7–C10–C11–C12A = -162.4 (5)° and C7–C10–C11–C13A = 58.6 (10)° for the minor component].

In the crystal packing, the molecules are linked into chains along the *b* axis by N—H···S hydrogen bonds (Fig. 2). These chains are cross-linked into a two dimensional network parallel to the *ab* plane by C—H···S hydrogen bonds (Table 1).

### Experimental

The title compound was prepared by following the literature procedure (Karthikeyan *et al.*, 2007). The solid product obtained was collected by filtration, washed with ethanol and dried. Colourless single crystals suitable for X-ray analysis were obtained from an ethanol solution by slow evaporation (yield 61%; m.p. 423–424 K).

### Refinement

The methylpropyl group is disordered over two orientations with refined occupancies of 0.685 (8) and 0.315 (8). During refinement, bond distances involving C12, C13, C12A and C13A atoms were restrained to 1.530 (7) Å, and their displacement parameters were restrained to an approximate isotropic behaviour. H atoms attached to N4 were located in a difference map and refined freely. The remaining H atoms were placed in calculated positions [N-H = 0.88 Å, C-H = 0.95-1.00 Å] and refined using a riding-model with *U*_iso_(H) = 1.5*U*_eq_(C_methyl_) and 1.2*U*_eq_(C,N).

### Crystal data

**Table:**

C_14_H_20_N_4_S	*F*_000_ = 296
*M**_r_* = 276.41	*D*_x_ = 1.227 Mg m^−^^3^
Monoclinic, *P*2_1_	Melting point = 423–424 K
Hall symbol: P 2yb	Mo *K*α radiation λ = 0.71073 Å
*a* = 5.9720 (3) Å	Cell parameters from 3612 reflections
*b* = 8.5153 (5) Å	θ = 2.8–30.0º
*c* = 14.8271 (6) Å	µ = 0.21 mm^−^^1^
β = 97.223 (3)º	*T* = 100.0 (1) K
*V* = 748.03 (7) Å^3^	Block, colourless
*Z* = 2	0.58 × 0.39 × 0.13 mm

### Data collection

**Table:**

Bruker SMART APEXII CCD area-detector diffractometer	3612 independent reflections
Radiation source: fine-focus sealed tube	3295 reflections with *I* > 2σ(*I*)
Monochromator: graphite	*R*_int_ = 0.029
Detector resolution: 8.33 pixels mm^-1^	θ_max_ = 30.0º
*T* = 100.0(1) K	θ_min_ = 2.8º
ω scans	*h* = −8→8
Absorption correction: multi-scan(SADABS; Bruker, 2005)	*k* = −11→11
*T*_min_ = 0.888, *T*_max_ = 0.974	*l* = −20→20
10373 measured reflections	

### Refinement

**Table:**

Refinement on *F*^2^	Hydrogen site location: inferred from neighbouring sites
Least-squares matrix: full	H atoms treated by a mixture of independent and constrained refinement
*R*[*F*^2^ > 2σ(*F*^2^)] = 0.046	*w* = 1/[σ^2^(*F*_o_^2^) + (0.0623*P*)^2^ + 0.2342*P*] where *P* = (*F*_o_^2^ + 2*F*_c_^2^)/3
*wR*(*F*^2^) = 0.119	(Δ/σ)_max_ = 0.001
*S* = 1.07	Δρ_max_ = 0.74 e Å^−^^3^
3612 reflections	Δρ_min_ = −0.54 e Å^−^^3^
199 parameters	Extinction correction: none
29 restraints	Absolute structure: Flack (1983), 1293 Friedel pairs
Primary atom site location: structure-invariant direct methods	Flack parameter: 0.05 (9)
Secondary atom site location: difference Fourier map	

### Special details

**Table:**

Experimental. The low-temperature data was collected with the Oxford Cyrosystem Cobra low-temperature attachment.
Geometry. All e.s.d.'s (except the e.s.d. in the dihedral angle between two l.s. planes) are estimated using the full covariance matrix. The cell e.s.d.'s are taken into account individually in the estimation of e.s.d.'s in distances, angles and torsion angles; correlations between e.s.d.'s in cell parameters are only used when they are defined by crystal symmetry. An approximate (isotropic) treatment of cell e.s.d.'s is used for estimating e.s.d.'s involving l.s. planes.
Refinement. Refinement of *F*^2^ against ALL reflections. The weighted *R*-factor *wR* and goodness of fit *S* are based on *F*^2^, conventional *R*-factors *R* are based on *F*, with *F* set to zero for negative *F*^2^. The threshold expression of *F*^2^ > σ(*F*^2^) is used only for calculating *R*-factors(gt) *etc*. and is not relevant to the choice of reflections for refinement. *R*-factors based on *F*^2^ are statistically about twice as large as those based on *F*, and *R*- factors based on ALL data will be even larger.

### Fractional atomic coordinates and isotropic or equivalent isotropic displacement parameters (Å^2^)

**Table:**

	*x*	*y*	*z*	*U*_iso_*/*U*_eq_	Occ. (<1)
S1	0.52913 (8)	0.63928 (8)	0.56907 (3)	0.02823 (14)	
N1	0.3013 (3)	0.7737 (3)	0.41731 (11)	0.0254 (4)	
H1	0.3880	0.8576	0.4194	0.030*	
N2	0.1200 (3)	0.7497 (3)	0.35093 (12)	0.0268 (4)	
N3	0.1622 (3)	0.5540 (3)	0.44853 (12)	0.0255 (4)	
N4	0.1075 (4)	0.4156 (3)	0.49036 (14)	0.0304 (5)	
H4A	0.205 (5)	0.349 (4)	0.477 (2)	0.036 (8)*	
H4B	0.120 (6)	0.435 (5)	0.551 (2)	0.050 (9)*	
C1	0.3316 (3)	0.6572 (3)	0.47770 (12)	0.0236 (4)	
C2	0.0397 (3)	0.6138 (3)	0.37174 (13)	0.0242 (5)	
C3	−0.1391 (4)	0.5226 (3)	0.31435 (13)	0.0262 (5)	
H3	−0.2464	0.4790	0.3546	0.031*	
C4	−0.0251 (3)	0.3868 (3)	0.27194 (13)	0.0245 (4)	
C5	−0.1039 (4)	0.2344 (3)	0.27769 (15)	0.0304 (5)	
H5	−0.2342	0.2154	0.3068	0.037*	
C6	0.0035 (4)	0.1101 (3)	0.24192 (15)	0.0299 (5)	
H6	−0.0538	0.0068	0.2468	0.036*	
C7	0.1950 (3)	0.1335 (4)	0.19865 (12)	0.0275 (4)	
C8	0.2718 (4)	0.2844 (4)	0.19190 (16)	0.0324 (6)	
H8	0.4009	0.3031	0.1620	0.039*	
C9	0.1650 (4)	0.4104 (3)	0.22788 (15)	0.0291 (5)	
H9	0.2220	0.5137	0.2224	0.035*	
C10	0.3109 (4)	−0.0036 (4)	0.15975 (16)	0.0368 (6)	
H10A	0.2910	−0.0980	0.1969	0.044*	
H10B	0.4747	0.0186	0.1643	0.044*	
C11	0.2221 (5)	−0.0394 (6)	0.0603 (2)	0.0730 (14)	
H11A	0.2458	0.0624	0.0293	0.088*	0.685 (8)
H11B	0.0645	−0.0562	0.0639	0.088*	0.315 (8)
C12	0.3694 (11)	−0.1501 (9)	0.0159 (4)	0.0661 (18)	0.685 (8)
H12A	0.3053	−0.1668	−0.0475	0.099*	0.685 (8)
H12B	0.3782	−0.2507	0.0483	0.099*	0.685 (8)
H12C	0.5210	−0.1053	0.0179	0.099*	0.685 (8)
C13	−0.0227 (7)	−0.0626 (7)	0.0405 (3)	0.0514 (15)	0.685 (8)
H13A	−0.0626	−0.0822	−0.0247	0.077*	0.685 (8)
H13B	−0.1010	0.0318	0.0578	0.077*	0.685 (8)
H13C	−0.0679	−0.1528	0.0750	0.077*	0.685 (8)
C12A	0.309 (2)	−0.2128 (11)	0.0475 (8)	0.057 (3)	0.315 (8)
H12D	0.2612	−0.2475	−0.0149	0.085*	0.315 (8)
H12E	0.2456	−0.2831	0.0901	0.085*	0.315 (8)
H12F	0.4742	−0.2149	0.0595	0.085*	0.315 (8)
C13A	0.220 (3)	0.0688 (16)	−0.0169 (8)	0.096 (6)	0.315 (8)
H13D	0.1648	0.1717	−0.0002	0.144*	0.315 (8)
H13E	0.1214	0.0271	−0.0691	0.144*	0.315 (8)
H13F	0.3740	0.0797	−0.0329	0.144*	0.315 (8)
C14	−0.2708 (4)	0.6302 (4)	0.24338 (15)	0.0321 (5)	
H14A	−0.3404	0.7156	0.2743	0.048*	
H14B	−0.3886	0.5693	0.2069	0.048*	
H14C	−0.1674	0.6742	0.2037	0.048*	

### Atomic displacement parameters (Å^2^)

**Table:**

	*U*^11^	*U*^22^	*U*^33^	*U*^12^	*U*^13^	*U*^23^
S1	0.0253 (2)	0.0327 (3)	0.0260 (2)	0.0030 (3)	0.00040 (16)	−0.0015 (2)
N1	0.0251 (8)	0.0242 (11)	0.0263 (8)	0.0010 (8)	0.0010 (6)	−0.0027 (7)
N2	0.0252 (9)	0.0283 (11)	0.0260 (8)	0.0019 (9)	0.0003 (6)	−0.0031 (7)
N3	0.0222 (8)	0.0301 (11)	0.0246 (8)	0.0005 (8)	0.0050 (6)	0.0010 (7)
N4	0.0269 (9)	0.0345 (12)	0.0308 (9)	−0.0024 (10)	0.0079 (7)	0.0085 (9)
C1	0.0222 (8)	0.0256 (13)	0.0239 (8)	0.0026 (10)	0.0068 (6)	−0.0016 (8)
C2	0.0227 (8)	0.0280 (14)	0.0224 (8)	0.0040 (10)	0.0045 (6)	0.0018 (8)
C3	0.0204 (9)	0.0301 (13)	0.0286 (9)	−0.0016 (10)	0.0044 (7)	0.0001 (9)
C4	0.0204 (9)	0.0295 (12)	0.0227 (8)	−0.0003 (10)	−0.0001 (7)	0.0000 (8)
C5	0.0283 (11)	0.0333 (14)	0.0312 (10)	−0.0001 (11)	0.0095 (8)	0.0065 (9)
C6	0.0326 (10)	0.0269 (14)	0.0302 (9)	−0.0007 (11)	0.0040 (8)	0.0039 (9)
C7	0.0235 (8)	0.0358 (12)	0.0220 (7)	0.0045 (13)	−0.0018 (6)	−0.0020 (11)
C8	0.0244 (10)	0.0444 (16)	0.0288 (10)	−0.0076 (11)	0.0054 (8)	−0.0084 (10)
C9	0.0263 (10)	0.0313 (13)	0.0304 (10)	−0.0074 (11)	0.0062 (8)	−0.0041 (9)
C10	0.0319 (12)	0.0444 (17)	0.0325 (11)	0.0092 (13)	−0.0017 (9)	−0.0107 (11)
C11	0.0469 (17)	0.120 (4)	0.0501 (17)	0.028 (2)	−0.0016 (13)	−0.044 (2)
C12	0.072 (4)	0.077 (4)	0.053 (3)	0.008 (3)	0.028 (3)	−0.029 (3)
C13	0.047 (2)	0.071 (4)	0.0333 (18)	−0.003 (2)	−0.0046 (16)	−0.018 (2)
C12A	0.040 (5)	0.092 (8)	0.040 (5)	−0.005 (5)	0.006 (4)	−0.021 (5)
C13A	0.086 (8)	0.107 (10)	0.090 (8)	0.008 (7)	−0.008 (6)	−0.040 (7)
C14	0.0249 (9)	0.0328 (13)	0.0368 (10)	−0.0003 (13)	−0.0027 (7)	0.0012 (11)

### Geometric parameters (Å, °)

**Table:**

S1—C1	1.688 (2)	C10—C11	1.533 (4)
N1—C1	1.334 (3)	C10—H10A	0.99
N1—N2	1.384 (2)	C10—H10B	0.99
N1—H1	0.8800	C11—C13	1.468 (4)
N2—C2	1.304 (3)	C11—C13A	1.468 (7)
N3—C1	1.368 (3)	C11—C12	1.497 (5)
N3—C2	1.372 (3)	C11—C12A	1.584 (7)
N3—N4	1.390 (3)	C11—H11A	1.00
N4—H4A	0.85 (4)	C11—H11B	0.96
N4—H4B	0.91 (3)	C12—H12A	0.98
C2—C3	1.496 (3)	C12—H12B	0.98
C3—C4	1.518 (3)	C12—H12C	0.98
C3—C14	1.535 (3)	C13—H13A	0.98
C3—H3	1.00	C13—H13B	0.98
C4—C5	1.386 (4)	C13—H13C	0.98
C4—C9	1.394 (3)	C12A—H12D	0.98
C5—C6	1.378 (4)	C12A—H12E	0.98
C5—H5	0.95	C12A—H12F	0.98
C6—C7	1.394 (3)	C13A—H13D	0.98
C6—H6	0.95	C13A—H13E	0.98
C7—C8	1.372 (4)	C13A—H13F	0.98
C7—C10	1.508 (4)	C14—H14A	0.98
C8—C9	1.389 (4)	C14—H14B	0.98
C8—H8	0.95	C14—H14C	0.98
C9—H9	0.95		
			
C1—N1—N2	113.3 (2)	C13—C11—C10	115.8 (3)
C1—N1—H1	123.4	C13A—C11—C10	126.3 (7)
N2—N1—H1	123.4	C12—C11—C10	113.4 (3)
C2—N2—N1	103.96 (18)	C13—C11—C12A	100.6 (5)
C1—N3—C2	108.67 (19)	C13A—C11—C12A	117.5 (8)
C1—N3—N4	127.56 (18)	C10—C11—C12A	102.8 (5)
C2—N3—N4	123.66 (19)	C13—C11—H11A	102.6
N3—N4—H4A	105 (2)	C12—C11—H11A	102.6
N3—N4—H4B	107 (3)	C10—C11—H11A	102.6
H4A—N4—H4B	112 (3)	C12A—C11—H11A	133.3
N1—C1—N3	103.48 (17)	C13A—C11—H11B	103.0
N1—C1—S1	128.94 (19)	C12—C11—H11B	124.4
N3—C1—S1	127.58 (18)	C10—C11—H11B	101.5
N2—C2—N3	110.60 (19)	C12A—C11—H11B	101.9
N2—C2—C3	125.67 (19)	H11A—C11—H11B	110.5
N3—C2—C3	123.2 (2)	C11—C12—H12A	109.5
C2—C3—C4	107.83 (17)	C11—C12—H12B	109.5
C2—C3—C14	110.3 (2)	H12A—C12—H12B	109.5
C4—C3—C14	112.86 (18)	C11—C12—H12C	109.5
C2—C3—H3	108.6	H12A—C12—H12C	109.5
C4—C3—H3	108.6	H12B—C12—H12C	109.5
C14—C3—H3	108.6	C11—C13—H13A	109.5
C5—C4—C9	117.8 (2)	H11B—C13—H13A	133.0
C5—C4—C3	120.96 (19)	C11—C13—H13B	109.5
C9—C4—C3	121.2 (2)	H11B—C13—H13B	100.7
C6—C5—C4	121.2 (2)	H13A—C13—H13B	109.5
C6—C5—H5	119.4	C11—C13—H13C	109.5
C4—C5—H5	119.4	H11B—C13—H13C	92.6
C5—C6—C7	121.0 (3)	H13A—C13—H13C	109.5
C5—C6—H6	119.5	H13B—C13—H13C	109.5
C7—C6—H6	119.5	C11—C12A—H12D	109.5
C8—C7—C6	117.9 (3)	C11—C12A—H12E	109.5
C8—C7—C10	121.6 (2)	H12D—C12A—H12E	109.5
C6—C7—C10	120.5 (3)	C11—C12A—H12F	109.5
C7—C8—C9	121.4 (2)	H12D—C12A—H12F	109.5
C7—C8—H8	119.3	H12E—C12A—H12F	109.5
C9—C8—H8	119.3	C11—C13A—H13D	109.5
C8—C9—C4	120.6 (2)	C11—C13A—H13E	109.5
C8—C9—H9	119.7	H13D—C13A—H13E	109.5
C4—C9—H9	119.7	C11—C13A—H13F	109.5
C7—C10—C11	113.7 (2)	H13D—C13A—H13F	109.5
C7—C10—H10A	108.8	H13E—C13A—H13F	109.5
C11—C10—H10A	108.8	C3—C14—H14A	109.5
C7—C10—H10B	108.8	C3—C14—H14B	109.5
C11—C10—H10B	108.8	H14A—C14—H14B	109.5
H10A—C10—H10B	107.7	C3—C14—H14C	109.5
C13—C11—C13A	91.1 (8)	H14A—C14—H14C	109.5
C13—C11—C12	116.9 (4)	H14B—C14—H14C	109.5
C13A—C11—C12	90.0 (8)		
			
C1—N1—N2—C2	−0.4 (2)	C2—C3—C4—C9	−49.2 (3)
N2—N1—C1—N3	0.1 (2)	C14—C3—C4—C9	72.9 (3)
N2—N1—C1—S1	−179.58 (16)	C9—C4—C5—C6	0.7 (3)
C2—N3—C1—N1	0.3 (2)	C3—C4—C5—C6	−177.8 (2)
N4—N3—C1—N1	−175.8 (2)	C4—C5—C6—C7	−0.1 (3)
C2—N3—C1—S1	179.98 (16)	C5—C6—C7—C8	−0.7 (3)
N4—N3—C1—S1	3.8 (3)	C5—C6—C7—C10	−180.0 (2)
N1—N2—C2—N3	0.6 (2)	C6—C7—C8—C9	0.8 (3)
N1—N2—C2—C3	−171.3 (2)	C10—C7—C8—C9	−179.9 (2)
C1—N3—C2—N2	−0.6 (2)	C7—C8—C9—C4	−0.2 (4)
N4—N3—C2—N2	175.7 (2)	C5—C4—C9—C8	−0.6 (3)
C1—N3—C2—C3	171.53 (18)	C3—C4—C9—C8	177.9 (2)
N4—N3—C2—C3	−12.1 (3)	C8—C7—C10—C11	−88.8 (3)
N2—C2—C3—C4	105.2 (3)	C6—C7—C10—C11	90.4 (3)
N3—C2—C3—C4	−65.8 (2)	C7—C10—C11—C13	−53.8 (5)
N2—C2—C3—C14	−18.5 (3)	C7—C10—C11—C13A	58.6 (10)
N3—C2—C3—C14	170.53 (19)	C7—C10—C11—C12	167.1 (4)
C2—C3—C4—C5	129.3 (2)	C7—C10—C11—C12A	−162.4 (5)
C14—C3—C4—C5	−108.6 (2)		

### Hydrogen-bond geometry (Å, °)

**Table:**

*D*—H···*A*	*D*—H	H···*A*	*D*···*A*	*D*—H···*A*
N1—H1···S1^i^	0.88	2.45	3.272 (3)	155
N4—H4A···S1^ii^	0.85 (3)	2.54 (3)	3.392 (3)	176 (3)
C5—H5···S1^iii^	0.95	2.78	3.704 (2)	165

Symmetry codes: (i) −*x*+1, *y*+1/2, −*z*+1; (ii) −*x*+1, *y*−1/2, −*z*+1; (iii) −*x*, *y*−1/2, −*z*+1.
